# 

**Published:** 1896-04

**Authors:** 


### EDITORIAL.

### Alabama State Dental Association.

The twenty-seventh annual meeting of this Association will be held at the Hotel Albert, Selma, Ala., April 14th to 17th, 1896. A very interesting and attractive programme has been prepared which embraces a number of important subjects that will doubtless be ably presented in papers and by discussion. It is to be hoped that all the members of the Association will be present, and a cordial invitation is extended to all practitioners in good standing in the State to be present, and it is earnestly desired that, so far as is practicable, all such will become members and assist in making the Association more efficient than ever in furthering the interests of the profession. A cordial invitation is also extended to the members of the profession outside of the State. Such are always welcome, and invariably add to the interest of the occasion.

The third day will be devoted to clinics and the more practical professional matters. The usual reductions by railroads and hotels have been procured.

The State Board of Dental Examiners will meet at the same time and place as the Association. It is very desirable that all who are especially interested in this meeting of the Board will be present, and thereby place themselves in a correct, legal position.

### American Medical Association—Dental Section.

The annual meeting of this body will be held at Atlanta, Ga., May 5–8, 1896. Preparations have been made which will insure a good meeting. The Section on Dental and Oral Surgery promises to be of unusual interest, and it is expected that a larger number than usual will be in attendance. We here give the titles of a portion of the papers that will be presented on that occasion:

Chairman's Address, R. R. Andrews, Cambridge, Mass.

"A Few of the Causes of Failures in the Dental and Medical Professions," B. B. Smith, Pensacola, Fla.

"Modern Methods of Treating the Antrum of Highmore," W. Xavier Sudduth, Chicago, Ills.

"The Teeth in Diagnosis," M. H. Fletcher, Cincinnati, O.

"Pyorrhœa Alveolaris," Eugene S. Talbot, Chicago, Ills.

### CONTENTS.

COMMUNICATIONS.

### THE DENTAL REGISTER,

**A MONTHLY MAGAZINE DEVOTED TO THE INTERESTS OF THE**

### DENTAL PROFESSION.

*Terms Reduced to* $2.00 *Per Year. Single Copies,* 25 *Cents.*

**EDITED BY PROF. J. TAFT, D.D.S.**

—AND—

**Published by SAM'L A. CROCKER & CO., Ohio Dental and Surgical Depot.**

The volume commences in January and closes in December.

The REGISTER being issued monthly is always fresh, therefore Indispensable to the practitioner who desires to keep posted up with the new inventions and improvements which are daily developed. Neither expense nor effort will be spared to give value and variety to its contents. Articles will be illustrated with well executed engravings whenever they will add to the interest or assist to explanation.

Its extensive circulation and frequency of issue make it one of the BEST DENTAL ADVERTISING MEDIUMS in the United States.

All subscriptions must begin with January or July.

Local or special notices, five lines or less, will be inserted for $3 each insertion; over five lines, 50 cts. per line.

All advertising bills payable in advance, or at furthest, after the issue of the first number containing the advertisement. All transient advertisements to be paid for in advance.

☞CONTRIBUTIONS SOLICITED.

Exclusively Editorial Communications should be addressed to the Editor.

The latest number of the REGISTER may be ordered with or without other goods at any time, and all letters should be addressed to [illegible]

